# Investigation of antimicrobial susceptibility patterns, risk factors and their impact on mortality in cancer patients at a tertiary care cancer hospital- A prospective study

**DOI:** 10.1186/s12941-024-00703-5

**Published:** 2024-06-26

**Authors:** Akshay Shelke, Pallavi Priya, Shiwani Mishra, Richa Chauhan, Krishna Murti, V. Ravichandiran, Sameer Dhingra

**Affiliations:** 1 Department of Pharmacy Practice, National Institute of Pharmaceutical Education and Research (NIPER) Hajipur, Dist. Vaishali, Bihar India; 2 Department of Microbiology, Mahavir Cancer Sansthan and Research Centre (MCSRC), Patna, Bihar India; 3 Department of Radiotherapy, Mahavir Cancer Sansthan and Research Centre (MCSRC), Patna, Bihar India

**Keywords:** Antimicrobial resistance, Cancer, 30-day mortality, Length of stay, Risk factors

## Abstract

**Background:**

Cancer patients are vulnerable to infections due to immunosuppression caused by cancer itself and its treatment. The emergence of antimicrobial-resistant bacteria further complicates the treatment of infections and increases the mortality and hospital stays. This study aimed to investigate the microbial spectrum, antimicrobial resistance patterns, risk factors, and their impact on clinical outcomes in these patients.

**Methods:**

A prospective study was conducted at a tertiary care cancer hospital in Patna, Bihar, India, which included cancer patients aged 18 years and older with positive microbial cultures.

**Results:**

This study analysed 440 patients, 53% (234) of whom were females, with an average age of 49.27 (± 14.73) years. A total of 541 isolates were identified, among which 48.01% (242) were multidrug resistant (MDR), 29.76% (150) were extensively drug resistant (XDR), and 19.84% (112) were sensitive. This study revealed that patients who underwent surgery, chemotherapy, were hospitalized, had a history of antibiotic exposure, and had severe neutropenia were more susceptible to MDR and XDR infections. The average hospital stays were 16.90 (± 10.23), 18.30 (± 11.14), and 22.83 (± 13.22) days for patients with sensitive, MDR, and XDR infections, respectively. The study also revealed overall 30-day mortality rate of 31.81% (140), whereas the MDR and XDR group exhibited 38.92% and 50.29% rates of 30-day mortality respectively (*P* < 0.001). Possible risk factors identified that could lead to mortality, were cancer recurrence, sepsis, chemotherapy, indwelling invasive devices such as foley catheter, Central venous catheter and ryles tube, MASCC score (< 21) and pneumonia.

**Conclusions:**

This study emphasizes the necessity for personalized interventions among cancer patients, such as identifying patients at risk of infection, judicious antibiotic use, infection control measures, and the implementation of antimicrobial stewardship programs to reduce the rate of antimicrobial-resistant infection and associated mortality and hospital length of stay.

**Supplementary Information:**

The online version contains supplementary material available at 10.1186/s12941-024-00703-5.

## Introduction

Antimicrobial resistance has become one of the most pressing global health threats, undermining the ability of antimicrobials to cure common infections, thereby worsening clinical outcomes with increased length of hospital stay, healthcare costs, morbidity and with 1.27 million and 4.95 million deaths attributable to and associated with bacterial AMR, respectively in 2019 [1, 2]. The World Health Organization ranks AMR as one of the top ten global threats to public health, projecting it to cause 10 million deaths annually by 2050. The risk assessment surveys of WHO have projected 389,000 deaths attributed to AMR in South Asia [3]. Specifically in India, there were 2,97,000 deaths caused by drug-resistant bacteria, with an additional 1,042,500 deaths linked to these infections [4].

Cancer patients, on the other hand, are at greater risk of developing various infections that can lead to worse outcomes and have a three-times greater risk of dying from a fatal infection than a patient without cancer because of their immunocompromised state due to the disease itself and the treatment modalities [5]. The infections in cancer patients caused by various pathogens are becoming extensively resistant to antimicrobials, which threatens recent advancements in cancer management [6, 7]. Unfortunately, these infections often require healthcare professionals to postpone or withhold intended cancer treatment, which further worsens the prognosis of cancer patients. Furthermore, antimicrobial resistance infections are the cause of more than half of all deaths in this patient [5]. According to a meta-analysis of pathogens isolated from post-chemotherapy infections, 26.8% of them were found to be resistant to the prophylactic antibiotics. Also, the study predicted that patients undergoing chemotherapy for haematological malignancies in the United States would experience between 4000 and 10,000 more infections and 500–1000 more fatalities annually if antibiotic efficacy were to drop by 30–70% [8]. Therefore, understanding the evolving epidemiology of bacterial and fungal infections and their sensitivity patterns in cancer patients is essential for developing effective prophylactic measures.

Despite the growing concern about antimicrobial resistance (AMR) and its impact on patient outcomes, there is a significant gap in the existing literature with limited understanding regarding impact of the antimicrobial resistance on clinical outcomes in terms of mortality, morbidity, length of hospital stays along with identification of patient-specific risk factors responsible for antimicrobial resistance and mortality with long term survival analysis among the cancer patients of low and middle countries including India [9–12].

This study aimed to investigate the susceptibility of microorganisms commonly isolated from cancer patients to antimicrobial agents, as well as the risk factors responsible for antimicrobial resistance. Furthermore, this study aimed to evaluate the impact of infection with resistant pathogens on the length of hospital stays and 30-day mortality.

## Materials and methods

### Patients, setting, data collection and study design

A prospective study was conducted on cancer patients at the Mahavir Cancer Sansthan and Research Centre in Patna, Bihar, India. This is a regional tertiary care cancer hospital in Bihar. Patients admitted to the inpatient department (IPD) with a first positive microbial culture and antimicrobial sensitivity, irrespective of the type and stage of cancer, were included in the study. Patients under the age of 18 with subsequent positive microbial cultures, those who were lost to follow-up and refused to give written informed consent, or who were not willing to participate in the study were excluded. The study was carried out on a total of 440 patients for 9 months from October 2022 to June 2023, and for all patients who met the inclusion criteria, demographic and clinical data relevant to this study were collected from case files of patients with positive microbial cultures and were followed up for 30 days from the date of culture sensitivity reports for 30-day mortality.

### Definition

BSI referred as to at least detection of one pathogenic microorganism in blood culture, if detected in urine it was referred as UTI (10,13). Hospital-acquired or nosocomial infection was defined as a positive culture obtained on day ≥ 3–48 h after hospital admission. All other infections were defined as being community-acquired [14]. Polymicrobial infections were defined as the isolation of > 1 bacterial species from the culture [12]. According to the classification of the National Cancer Institute (NCI), neutropenia was defined as an absolute neutrophil count (ANC) falling below 1500 neutrophils/mm3, with its severity categorized as follows: Category-I (ANC ≥ 1500–≤2000), Mild/Category-II (ANC 1000–1500 neutrophils/mm3), Moderate/Category-III (500–1000 neutrophils/mm3), and Severe/Category-IV (< 500 neutrophils/mm3) [15]. Neutropenic fever was characterized as a single oral temperature exceeding 38.3 °C (101 °F) or a temperature higher than 38 °C (100.4 °F) sustained for at least one hour, with an accompanied ANC < 1500 neutrophils/mm^3^ [15, 16]. The MASCC risk index was calculated using the following variables: burden of illness, blood pressure, presence or absence of chronic obstructive pulmonary disease, solid tumour or haematological malignancy with or without a history of previous fungal infection, dehydration, inpatient or outpatient status at the time of onset of neutropenic fever, and age, with each variable having a score between 0 and 3. Higher scores indicate lower risk, with a maximum of 26 points. Using a cutoff value of ≥ 21 points differentiates patients with low risk from those with high risk (< 21 points) for serious complications of febrile neutropenia, e.g., death, admission to the intensive care unit, or hypotension [16].

A positive bacterial culture was considered as multidrug resistant (MDR) when at least one antibiotic from three or more classes of antibiotics were resistant to isolates, whereas those bacterial isolates that were non-susceptible to all antibiotics except for two or fewer class of antibiotics were referred as extremely drug resistant (XDR) [17]. 30-day mortality was evaluated starting from the day of antibiotic susceptibility testing findings to analyze long-term survival and post discharge mortality in individuals with resistant infections [8, 17].

### Microbiological investigations and antibiotic sensitivity testing

According to the hospital’s established policy, relevant samples such as blood, urine, pus/wound swabs, sputum, bronchoalveolar lavage, pleural fluid, and stool were obtained from various clinical areas. All samples were processed following accepted microbiology laboratory practices. Susceptibility testing was carried out according to the 2021 Clinical Laboratory Standards Institute (CLSI) criteria. All the positive microbial cultures were correlated with signs and symptoms of infections of patients by the physicians and cultures with colonization were excluded from the study. Antibiotic susceptibility testing was performed using the Kirby–Bauer disc diffusion technique. The respective organisms were cultured on Mueller Hinton agar media. Antibiotic discs with the required strengths were placed on the surface of the inoculated media and then incubated overnight. The zones of inhibition were measured the following day and compared with the CLSI interpretive zone diameter to classify them as sensitive, intermediate, or resistant. *Staphylococcus aureus* (ATCC 25,923), *Escherichia coli* (ATCC 25,922) and *Pseudomonas aeruginosa* (ATCC 27,853) were used for quality control [9].

### Fungal identification and susceptibility testing

All the samples were processed following conventional mycological procedures, including microscopy and culture. For microscopy, a KOH mount and gram stain were prepared from samples received in the microbiology department. Culture was done on Sabouraud Dextrose Agar (SDA), and identification of the fungus (moulds) was confirmed on the basis of growth characteristics and morphology on the LPCB (lactophenol cotton blue stain) mount. For yeast and yeast-like cells, SDA CHROM agar (HiMedia, India) and cornmeal agar were used. In the case of candida, a germ tube test was done, and speciation was confirmed by HiCHROM agar and chlamydospore formation on cornmeal agar [18, 19].

Antifungal susceptibility testing was performed using the disk diffusion method according to Clinical Laboratory Standards Institute (CLSI)-approved standards M-60 and M-44. Amphotericin B, Itraconazole, Fluconazole, Voriconazole, and Posaconazole were tested for antifungal susceptibility [19].

### Statistical analysis

The independent sample t test and Kruskal‒Wallis test was used to compare continuous variables according to whether they followed a normal or nonnormal distribution. The chi-square test or Fisher’s exact test was used to analyse differences in 30-day survival and 30-day mortality group respectively for categorical variables. A bivariate logistic regression model was used to develop a mortality predictor model. A multinomial logistic regression was carried out to predict the factors that may lead to antimicrobial resistance in cancer patients, and odds ratios (ORs) with confidence intervals (95% CIs) were calculated. Kaplan-Meier survival analysis was used to estimate the overall survival (OS) rate. All tests of significance were two-tailed, with a *p* value less than 0.05 indicating statistical significance. These *p* values are shown in bold font. The data were analysed using the Statistical Package for the Social Sciences (SPSS) Version 27.

## Results

### Study population and patient characteristics

The study included a total of 440 patients who had culture-positive reports for either bacteria or fungi. A total of 234 (43.3%) patients were female. The mean age of the patients was 49.48 years (± 14.73). The majority of the patients (*n* = 375; 85%) had solid tumour, while the rest had haematological cancer. (Table 1)

**Table 1:** Comparison of baseline characteristics between the 30-day survival and 30-day mortality subgroup

Characteristics	Total (*n* = 440) (%)	30-day survival (*n* = 300) (%)	30-day mortality (*n* = 140) (%)	*p* value*
**Age (Years)**	49.48 (± 14.73)	49.91(± 14.85)	48.87(± 14.95)	0.109
**Gender**				0.120
Male Female	206(46.82) 234(53.18)	133 (44.33) 167(55.66)	73(52.14) 67(47.85)	
**Cancer status**				0.352
Recent Progression Recurrence Remission	255(47.10) 84(15.50) 66(12.25) 35(6.55)	181(60.36) 44(14.72) 41(13.73) 34(11.37)	74(52.92) 40(28.64) 25(17.93) 1(0.71)	
**Types of cancer**				0.018*
Solid tumour Haematological	375(85.22) 65(14.88)	264(88.00) 36(12.00)	111(79.30) 29(20.70)	
**Stages of cancer**				< 0.001*
I II III IV Unknown	27(6.10) 157(35.70) 146(33.20) 42(9.50) 68(15.50)	26(8.72) 114(38.00) 100(33.38) 22(7.33) 38(12.77)	1(0.74) 43(30.76) 46(32.90) 20(14.31) 30(21.49)	
**Histological classification**				0.217
Carcinoma Leukaemia Sarcoma Myeloma Lymphoma Mixed types Melanoma	341(77.56) 55(12.52) 18(4.12) 11(2.50) 07(1.64) 04(1.00) 03(0.86)	242(80.73) 29(9.74) 11(1.33) 09(3.72) 04(3.09) 01(1.010) 03(0.30)	99(70.70) 26(18.68) 07(5.01) 03(2.11) 03(2.13) 02(1.47) 00(0)	
**Comorbidities**				0.201
Hypertension Diabetes mellitus Hypertension + Diabetes Hypothyroidism	57(12.95) 37(08.40) 44(10.00) 46(10.45%)	40(13.39) 27(09.00) 26(8.71) 34(11.33)	17(25.02) 10(07.18) 18(12.85) 12(8.57)	
**History (within 3 months)**				
Surgery Radiation Chemotherapy	129(29.33) 75(17.00) 164(37.37)	88(29.32) 49(16.00) 109(36.00)	41(29.28) 26(18.57) 55(39.28)	0.922 0.589 0.494
**Treatment received**				0.004*
Chemotherapy alone Radiotherapy alone Surgery alone Chemotherapy + radiotherapy Other	148(33.60) 24(5.50) 116(26.40) 26(5.90) 126(28.64)	98(32.72) 16(5.38) 93(31.00) 21(7.01) 72(24.09)	50(35.76) 8(5.72) 23(16.42) 05(3.64) 54(37.86)	
**Invasive devices**				
Foley catheter Ryle’s tube Central venous catheter	274(62.35) 143(32.55) 33(7.50)	162(59.12) 69(15.68) 10 (2.72)	112(40.88) 74(16.81) 23(5.22)	< 0.001* < 0.001* < 0.001*
**NCI category**				< 0.001*
0 I II III IV Neutrophilia	140(31.82) 12(2.75) 7(1.64) 11(2.52) 41(9.302) 229(52.03)	117(39.23) 8(2.72) 5(1.78) 8(2.70) 21(7.00) 141(47.45)	23(16.47) 4(2.92) 2(1.43) 3(2.12) 20(14.36) 88(62.85)	
**MASCC risk score**				< 0.001*
High risk (< 21) Low risk (> 21)	174(38.46) 266(59.12)	32(31.30) 103(65.30)	142(68.70) 163(34.70)	

*Categorical variables among survival and non-survival subgroups were compared using the Chi-square/Fisher’s exact test. Continuous variable was compared using independent sample t test. All significance tests were two-tailed, with a *p* value less than 0.05 indicating statistical significance, and such *p* values are shown as star in superscript

Most of the patients had head and neck cancer (56,12.5%), followed by cervical cancer (54,12.4%), acute leukaemia (41,9.6%), breast cancer (40,9.4%) and gallbladder cancer (39,9.1%). However, mortality was higher in patients with acute leukaemia (20, 14.28%), followed by gall bladder cancer (18, 12.58%), head and neck cancer (17, 12.14%) and cervical cancer (16, 11.42%). (Supplementary Table 1).

A total of 129 (29.3%) patients had a history of surgery within the last 3 months; 75 (17%) received radiotherapy; and 164 (37.3%) underwent chemotherapy. Approximately 148 (33.6%) patients received chemotherapy when they were admitted to the inpatient department, and 116 (26.4%) underwent surgery as a treatment for the management of cancer.

According to the classification of the National Cancer Institute for Neutropenia, 140 (31.81%) patients did not have any type of neutropenia, while 12 (2.7%), 7 (1.6%), 11 (2.5%), and 41 (9.30%) were in the categories of I, II (mild), III (moderate), and IV (severe), respectively. In Category IV (severe neutropenia), the patients’ absolute neutrophil count (ANC) was less than 500 cells/mm^3^, and approximately 48.78% of the patients died (*P* < 0.001). Additionally, 174 (38.4%) patients were in a high-risk class whose Multinational Association for Cancer Care (MASCC) risk score was less than 21.

### Microbiology

A total of 1148 samples were collected in the microbiology department for culture and antimicrobial susceptibility testing, of which 541 (47.82%) were culture positive,504 were bacterial, and the remaining were fungal. Approximately 76.98% of these samples were gram-negative bacteria, with *Klebsiella pneumoniae* (94, 17.4%), *Escherichia coli* (60, 11.1%), *Klebsiella oxytoca* (58, 10.7%), and *Pseudomonas aeruginosa (53, 10.5%)* being the most commonly isolated bacteria. The most common gram-positive bacteria were Methicillin-resistant *Staphylococcus aureus* (42, 7.8%) and *Enterococcus species. (40, 7.4%)*, and *coagulase-negative Staphylococcus (17, 3.1%).* Fungal infections involving *nonalbicans Candida* (3.9%) were more common than *infections involving Candida albicans* (3%).

When comparing the difference and frequency of gram-negative and gram-positive bacteria in the 30-day survival group to those in the 30-day mortality group, gram-negative bacteria contributed more to mortality (79.67%). The 30-day mortality was greater in cancer patients infected with *K. pneumoniae*, *K. oxytoca, P. aeruginosa* and *E. coli* from the gram-negative class of bacteria and *MRSA*, *Enterococcus spp*. and *Coagulase negative staphylococcus* (*CONS*) from the gram-positive class of bacteria (Table 2).

**Table 2:** Comparison of microbiological characteristics between survival and 30-day mortality subgroups

	Total number of isolates (*n* = 541) (%)	30-day survival (*n* = 359 isolates) (%)	30-day mortality (*n* = 182 isolates) (%)	*p* value
**Gram-negative bacteria**	388(71.72)	253(70.43)	145(79.67)	0.681
*Klebsiella pneumoniae*	94(17.37)	57(15.88)	37(20.03)	
*Escherichia coli*	60(11.09)	41(11.42)	19(10.43)	
*Klebsiella oxytoca*	58(10.72)	37(10.30)	21(11.53)	
*Pseudomonas aeruginosa*	53(9.79)	38 (10.58)	15(8.24)	
*Klebsiella.* spp	43(7.94)	24 (6.68)	14(7.69)	
*Acinetobacter baumannii*	24(4.43)	15 (4.17)	09(4.94)	
*Pseudomonas aeruginosa* Other	24(4.43) 33 (6.09)	16 (4.45) 23(6.00)	08(4.39) 10(5.01)	
**Gram-positive bacteria**	116(21.45)	84(23.59)	32(17.58)	
*MRSA*	42(7.82)	28(07.79)	14(7.69)	
*Enterococcus* spp.	40(7.41)	31(8.67)	09(4.94)	
*CoNs* Other	17(3.11) 16(3.00)	09(2.50) 12(3.25)	05(2.74) 04(2.17)	
**Fungi**	37 (6.83)	22(6.12)	15(8.24)	
*Candida albicans* *Non albicans candida*	16(2.95) 21(3.88)	11(3.06) 11(3.06)	05(2.71) 10(5.49)	
**Source of infection**				0.011*
Pus	189(34.93)	150(43.30)	39(7.73)	
Urine	186(34.47)	111(32.02)	75(14.88)	
Blood	84(15.56)	41(10.74)	43(8.55)	
Sputum	55(10.24)	39(0.33)	16(3.17)	
Tracheal aspirate Other	11(2.02) 16(0.92)	2(9.70) 14(10.41)	09(1.73) 00(00)	
**Type of bacterial infection**				0.004*
Monomicrobial Polymicrobial	351(79.77) 89(20.23)	248(82.77) 52(17.33)	103(73.57) 37(43.43)	
**Type of infection**				0.487
Community-acquired Nosocomial	134 (26.42) 407(75.28)	64(21.34) 227(75.76)	46(25.38) 136(74.72)	
**Type of bacteria**				0.653
Gram-negative bacteria Gram-positive bacteria	388(76.98) 116(23.02)	257(66.23) 80(68.96)	131(33.77) 36(31.04)	
**Antimicrobial susceptibility**				< 0.001*
Sensitive MDR XDR	100(23.52) 201(47.29) 124(29.17)	87(29.89) 148(50.85) 56(19.99)	13(9.70) 53(39.55) 68(50.76)	

The primary source of infection was nosocomial or hospital-acquired infection rather than community-acquired infection. Cancer patients also exhibited polymicrobial infection (89,20.2%), where they tested positive for more than one microorganism, regardless of the sample type. Polymicrobial infection was found to be significantly (*P* = 0.004) related to 30-day mortality (Table 2). The distribution of identified pathogens across several sites of microbial culture is shown in Table 3. *Pseudomonas aeruginosa* was the most common pathogen found in pus, whereas *Klebsiella pneumoniae* was predominant in urine, blood, sputum, and tracheal aspirate.

**Table 3:** Distribution of pathogens on the basis of source of infection (*n = 541)*

Source of infection	Pus (*n*-189)	Urine (*n*-186)	Blood (*n*-84)	Sputum (*n*-55)	Tracheal aspirate (*n*-11)	Bile (*n*-12)	Other* (*n*-4)
*Klebsiella pneumoniae*	27	33	16	10	5	2	1
*Klebsiella oxytoca*	10	30	7	9	1	2	0
*Klebsiella species*	18	16	6	1	0	2	0
*E. coli*	21	31	2	3		2	1
*Pseudomonas aeruginosa*	36	6	3	5	3	0	0
*Pseudomonas spp.*	10	8	1	3	0	2	0
*Acinetobacter baumannii*	9	8	7	0	1	0	0
*MRSA*	21	2	12	6	0	0	0
*MSSA*	2	2	5	1	0	0	0
*Enterococcus spp.*	8	19	10	3	0	0	0
*Citrobacter spp.*	10	3	2	0	0	1	0
*CoNS*	5	6	12	0	0	0	0
*Proteus Mirabilis*	7	5	0	0	0	0	0
*Candida albicans*	0	7	0	8	0	1	1
*Non- albicans candida*	1	11	1	6	1	0	1

*Others include- Drain fluid, Pleural fluid and stool samples

**Table 4:** Risk factor analysis

**A. Multinomial logistic regression for the prediction of drug resistance**				
Factors	Total	Odds Ratio	CI (95%)	*p*-Value
Multidrug resistance (MDR)^a^ (*n* = 201)				
History of surgery	48	2.73	(1.33–5.58)	0.006*
History of chemotherapy	71	4.95	(2.15–11.40)	0.000*
Recent antibiotic use	157	2.46	(1.74–6.48)	0.001*
Prior hospitalization	164	2.93	(1.08–7.92)	0.034*
Type of bacteria	50	23.44	(3.76-145.84)	0.001*
Neutropenia (ANC < 500)	08	1.24	(1.04–1.42)	0.009*
Extensively drug resistance (XDR)^a^ (*n* = 124)				
History of surgery	13	1.92	(1.56–4.53)	0.040*
History of chemotherapy	43	3.84	(1.48–9.96)	0.006*
Recent antibiotic use	106	3.43	(1.65–10.84)	0.021*
Prior hospitalization	103	3.87	(1.25-12.00)	0.019*
Neutropenia (ANC < 500)	05	1.34	(1.12–1.61)	0.001*
**B. Binomial logistic regression for predictor analysis of 30-day mortality**				
Cancer recurrence	66	27.65	(2.00-38.75)	0.013*
Sepsis	36(1.33)	6.79	(1.23–37.68)	0.028*
Chemotherapy	148(32.7)	3.54	(1.06–11.80)	0.039*
Foley catheter Ryles tube Central venous catheter	274(36.81) 143(15.68) 33 (2.72)	4.13 2.74 12.51	(1.72–9.92) (1.18–6.33) (2.85–54.96)	0.002* 0.018* < 0.001*
NCI category-III NCI category-IV Neutrophilia	11(2.7) 41(7.0) 228(47.0)	1.09 1.39 1.26	(0.08–14.23) (0.08–1.89) (0.11–0.63)	0.045 0.002 0.003
MASCC score (< 21)	174(31.3)	2.49	(1.08–5.73)	0.032*
*Klebsiella pneumoniae*	94(17.3)	1.40	(0.46–2.20)	0.040
Pneumonia	55(0.3)	2.10	(1.20–4.10)	0.018*
VAP	11(9.7)	2.35	(0.75–3.25)	0.032
Polymicrobial	89(17.3)	1.58	(0.36–2.35)	0.027
Community acquired	113(21.3)	1.53	(0.49–4.70)	0.012
MDR isolates XDR isolates	201(35.11) 124(13.09)	3.40 8.12	(2.12–5.44) (4.11–16.06)	< 0.001* < 0.001*

* *p* value less than 0.05 indicates statistical significance. Abbreviations: NCI- National Cancer Institute, MASCC- Multinational Association for Supportive Care in Cancer, VAP- Ventilator-associated pneumonia, MDR- Multidrug resistant, XDR- Extensively drug resistant

Fluoroquinolones, such as ciprofloxacin in combination with amoxicillin/clavulanic acid or clindamycin in place of amoxicillin/clavulanic acid in patients with penicillin allergy, were prescribed as empirical antibiotics based on the local prevalence of hospitalization and the suspected infection. In high-risk patients, antipseudomonal beta lactams such as cefepime, piperacillin-tazobactam, Amikacin, meropenem or imipenem, and cilastatin were used as empirical therapy.

### Antimicrobial susceptibility

**Fig. 1:**
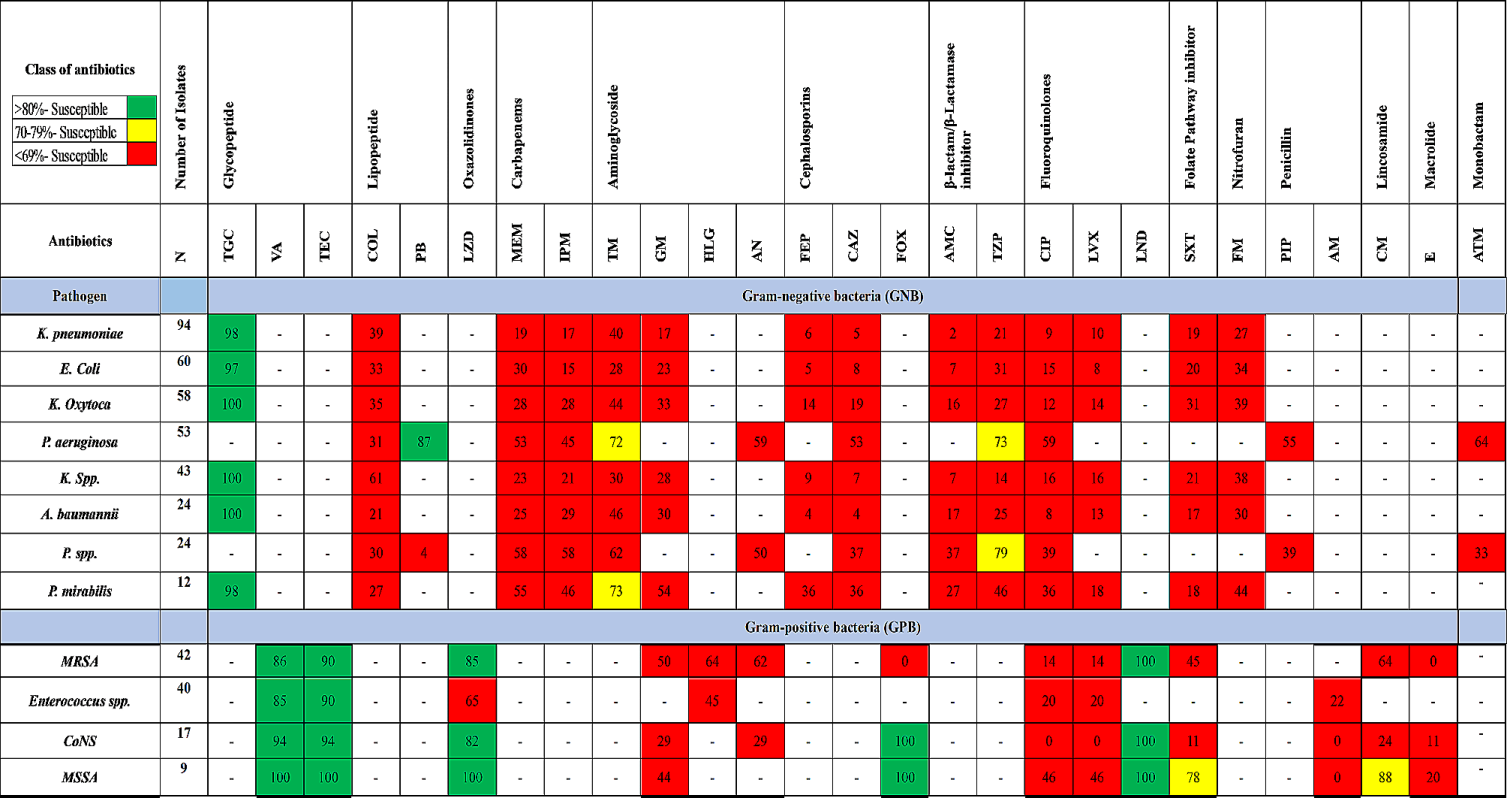
Antibiogram of predominant pathogens. **Antimicrobial agent abbreviations (CLSI Recommended), TGC = Tigecycline; VA = Vancomycin; TEC = Teicoplanin; CL = Colistin; PB = Polymixin-B; LZD = Linezolid; MEM = Meropenem; IPM = Imipenem; TM = Tobramycin; GM = Gentamicin; HLG = High-level gentamicin; AN = Amikacin; FEP = Cefepime; CAZ = Ceftazidime; FOX = Cefoxitin; AMC = Amoxicillin-clavulanate; TZP = Piperacillin-Tazobactam; CP = Ciprofloxacin; LVX = Levofloxacin; LND = Levonadafloxacin; STX = Trimethoprim-Sulfamethoxazole; FM = Nitrofurantoin; PIP = Piperacillin; AM = Ampicillin; CM-Clindamycin; E = Erythromycin; ATM = Aztreonam; *MRSA = Methicillin-resistant Staphylococcus aureus; MSSA = Methicillin-sensitive Staphylococcus aureus; CoNs-Coagulase-negative Staphylococcus aureus*

According to the World Health Organization classification for antibiotic susceptibility, the identified bacteria were placed in a particular class based on their susceptibility to antibiotics, such as sensitive, multidrug-resistant (MDR) or extensive drug-resistant (XDR) bacteria. Of the total bacterial isolates, 112 (19.84%) were sensitive to all antibiotics tested, whereas 242 (48.01%) bacteria were resistant to at least one antibiotic from three or more classes of antibiotics (MDR). Furthermore, 150 (29.76%) bacterial isolates were non-susceptible to all antibiotics except for two or fewer class of antibiotics (XDR) (Fig. 1). The distributions of sensitive, MDR and XDR bacteria with 30-day mortality for each antibiotic susceptibility class in individual cancer types are shown in Supplementary Table 1.

Most of the gram-negative bacteria were resistant to all the classes of antibiotics, including carbapenems, 3rd and 4th generation cephalosporins, aminoglycosides, fluoroquinolones, beta lactam and beta-lactamase inhibitors. The sensitivity patterns of the most common gram-negative bacterial isolates are shown in Supplementary Fig. 1, and those of the most common gram-positive bacterial isolates are shown in Supplementary Fig. 2.

Extensive drug-resistant organisms detected in the gram-negative category included *Klebsiella pneumoniae* 47 (31.3%), *E. coli* 30 (20%), *Klebsiella oxytoca* 23 (15.3%), *Klebsiella spp.*18 (12%) and *Acinetobacter baumannii* 12 (8%). Only one Coagulase negative *Staphylococcus* pathogen was an XDR bacterium from the gram-positive class. Common multidrug resistant organisms (MDROs) from gram-negative bacteria detected were *Klebsiella pneumoniae* 40(16.5%), *Klebsiella oxytoca* 28(11.6%), *E. coli* 26(10.7%), *Klebsiella spp*.22 (9.1%), and *Pseudomonas aeruginosa* 22 (9.1%). There were 36 (14.9%) MRSA strains, 17 (7%) Enterococcus species, and 12 (5%) CoNs (Fig. 1**).**

Compared with bacterial isolates, fungal isolates were more susceptible to antimicrobial drugs. 90% of the total non-*Candida albicans* isolates were sensitive to fluconazole and voriconazole, with 75% sensitivity to amphotericin-B, 65% to ketoconazole and 62% to itraconazole (Supplementary Fig. 3A), whereas 85% of the *Candida albicans* isolates were sensitive to voriconazole, 72% to fluconazole, 68% to amphotericin-B and ketoconazole, with a poor sensitivity of 52% for itraconazole and posaconazole (Supplementary Fig. 3B).

### Antimicrobial susceptibility classes and length of hospital stay

Independent samples Kruskal‒Wallis tests revealed a statistically significant difference between the three different classes of antibiotic resistance and hospital length of stay (*P* < 0.001). Additionally, there was a significant difference between the average length of stay of 16.90 days (± 10.232), 18.30 days (± 11.144), and 22.83 days (± 13.226) in patients infected with sensitive, MDR and XDR bacteria, respectively. Moreover, the analysis demonstrated that there was no statistically significant difference between the sensitive and MDR groups (*P*-0.305). In contrast, the differences between the sensitive-XDR group (*P* < 0.001) and MDR-XDR (*P* < 0.001) groups were highly statistically significant. (Supplementary Fig. 4)

### Risk factors for the development of antibiotic resistance in cancer patients

As shown in Table 4A, MDR isolates were more common in patients who had a history (within the last 3 months) of surgery (Odds Ratio [OR], 2.73; Confidence Interval [CI], 1.33–5.58) chemotherapy (OR, 4.95; CI, 2.15–11.40), or hospitalization (OR, 2.93; CI, 1.08–7.92) and who had used antibiotics (OR, 2.46; CI, 1.74–6.48) Additionally, these isolates were more prevalent in patients with severe neutropenia (OR, 1.24; CI, 1.04–1.42) and those infected with gram-negative bacteria (OR, 23.44; CI, 3.76-145.84). On the other hand, XDR isolates were more frequent in patients who had a history (within the last 3 months) of surgery (OR, 1.92; CI, 1.56–4.53), chemotherapy (OR, 3.84; CI, 1.48–9.96), or hospitalization (OR, 3.87; CI, 1.25-12.00) and who had used antibiotics (OR, 3.43; CI, 1.65–10.84), as well as in severe neutropenic patients (OR, 1.34; CI, 1.12–1.61). Supplementary Table 2 represents a multinomial logistic regression analysis for predicting the possible risk factors fostering antimicrobial resistance among cancer patients.

### Risk factors for overall 30-day mortality

Table 4B summarizes the potential risk factors for all-cause 30-day mortality in hospitalized cancer patients with positive microbial cultures. The variables associated with mortality were cancer recurrence (OR-27.65; CI, 2.00-38.75), sepsis (OR-6.790; CI, 1.23–37.68), chemotherapy (OR-3.546; CI, 1.06-11.805), the use of a Foley catheter (OR-4.131; CI, 1.720–9.925), ryles tube (OR-2.742; CI, 1.188–6.335), a central venous catheter (OR-12.512; CI, 2.854–54.96), a high MASCC risk score (OR-2.493; CI, 1.083–5.738), coinfection with pneumonia (OR-2.103; CI, 1.200–4.100), MDR bacteria (OR-3.401; CI, 2.125–5.447) and XDR bacteria (OR-8.122; CI, 4.118–16.067). Other variables included to assess the risk of 30-day mortality in cancer patients by using bivariate regression are shown in Supplementary Table 3.

### Subgroup analysis of 30-day mortality in cancer patients

Overall, the 30-day mortality rate was 140 (31.81%). Among the total deaths, 134 (95.71%) were due to bacterial infection, and the remaining deaths were due to fungal infection. Patients with nosocomial infections had a higher mortality rate (74.7%) than did those with community-acquired infections (*P* = 0.473). Monomicrobial infection was the major contributor to mortality, with 103 patients (56.6%), but the mortality rate among patients with polymicrobial infection was higher (41.57%) than that among patients with monomicrobial infection (*P* < 0.004). The mortality rate of patients with XDR infections was 50.29%, which was greater than that of patients with MDR infections (38.92%), and the lowest mortality rate was noted for patients infected with bacteria sensitive to all antibiotics. The overall difference between the survival and 30-day mortality groups on the basis of the class of antibiotic susceptibility was statistically significant (*P* < 0.001) (Table 2).

### Survival analysis in cancer patients

**Fig. 2:**
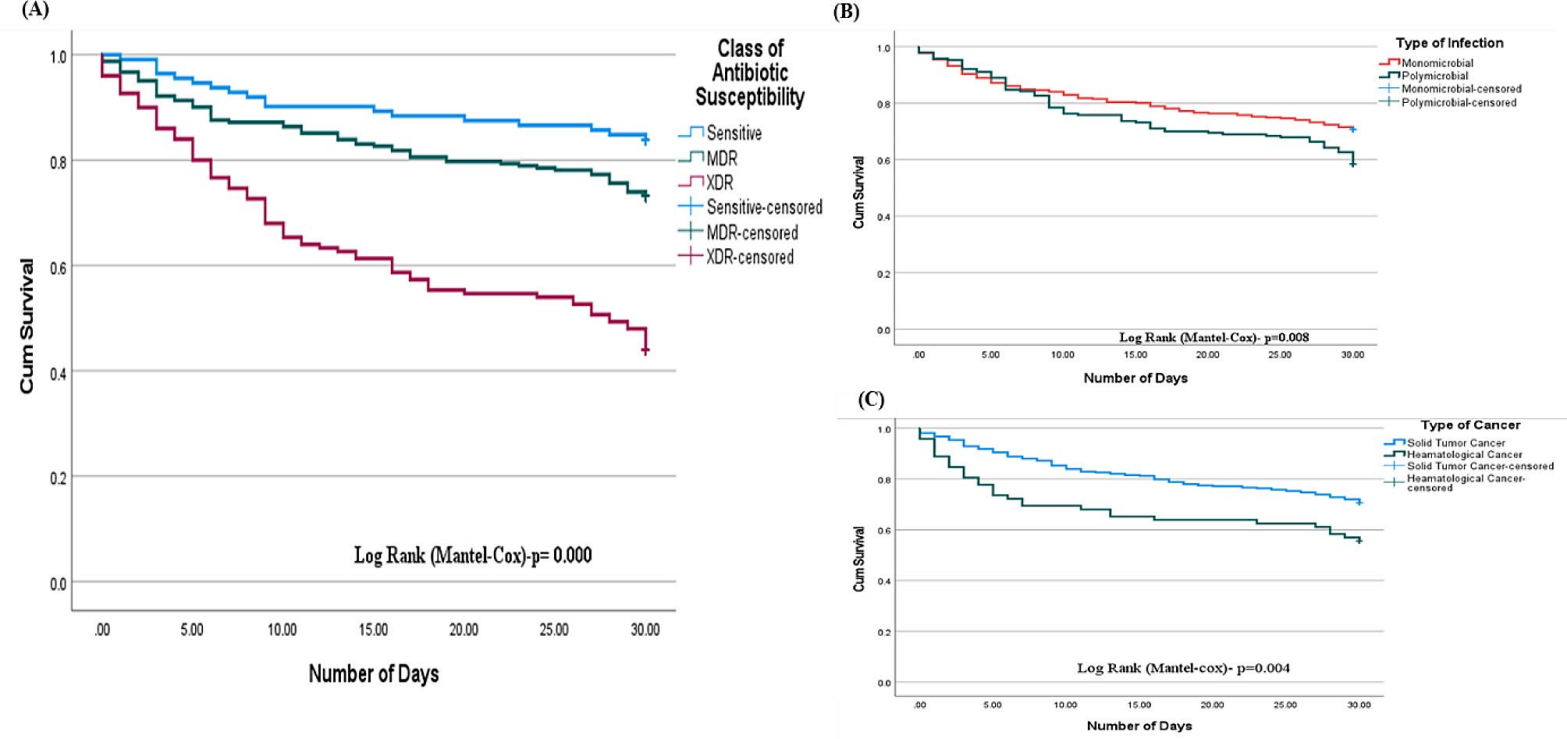
Kaplan meier survival curve. The 30–day survival rates are illustrated on the basis of antibiotic susceptibility class. The X-axis represents the number of days each patient was followed for survival analysis. The Y-axis indicates the cumulative survival. **(A)** Survival analysis among cancer patients based on antibiotic susceptibility class (sensitive, MDR, or XDR). **(B)** Illustration of the differences in survival between the monomicrobial and polymicrobial types of infection. **(C)** Comparison of survival between patients with solid tumors and patients with hematological cancer

Figure 2A shows the Kaplan‒Meier survival curves for all patients with sensitive, MDR, and XDR types of bacterial infections. The 30-day survival rate was lower for patients with XDR isolates (log-rank Mantel Cox *P* = 0.000) than for those with MDR and sensitive isolates. The Kaplan‒Meier curve comparing survival between patients with monomicrobial and polymicrobial infections revealed poorer survival in patients with polymicrobial infections than in patients with monomicrobial infections (log rank Mantel–Cox test, *P =* 0.008) (Fig. 2B**)**. Patients with haematological cancer had a poorer survival rate than patients with solid tumours (log rank Mantel–Cox test *P* = 0.000) (Fig. 2C**)**.

## Discussion

Previous studies conducted in India on cancer patients have focused mainly on the microbiological spectrum and antibiotic susceptibility pattern, and a few studies have reported clinical outcomes in terms of mortality. Therefore, there is a gap in understanding the potential risk factors fostering antimicrobial resistance as well as the possible factors responsible for mortality in cancer patients with infections, as well as the survival rate in Indian clinical settings.

The major findings of this study indicate that there is a significant difference in mortality among cancer patients who are positive for MDR and XDR pathogens (37.23%) compared to those who have an infection with antibiotic-sensitive bacteria (14%), which is significantly higher than the mortality rate reported from other studies [5, 20]. A multicentre study carried out in 10 hospitals in India found that patients infected with MDR and XDR bacteria had 1.57- and 2.65-times higher risk of mortality compared to patients with susceptible infections, with an overall mortality rate of 13.1%. The study also emphasized that cancer patients face a higher risk of death from resistant infections than non-cancer patients [21]. Additionally, out of the total isolates, 76.47% of the patients were culture positive for either MDR or XDR bacteria. The pathogens isolated from bacterial culture were predominantly gram-negative bacteria (GNB), supporting the recent trend suggesting a shift from gram-positive infections to gram-negative infections in cancer patients in India and across the globe [12, 22, 23]. The most common GNB isolated was *Klebsiella pneumoniae*, followed by *E. coli*, *Klebsiella oxytoca*, *Pseudomonas aeruginosa*, *Klebsiella spp.* and *Acinetobacter baumannii*. These results are consistent with those of other studies published in India [9, 11, 12]. However, one study from India reported a high prevalence of *Pseudomonas spp.*, followed by *E. coli* and *Klebsiella spp.* [14]. The major fungal isolate identified was non-*Candida albicans*, the findings are consistent with study conducted in immunocompromised patients where non- candida albicans was predominant fungi causing IFI [24]. Invasive fungal infections were more prevalent in cancer patients with solid tumours than in those with haematological malignancies; these findings contrast with those of studies conducted in Spain, where all the fungal isolates detected were from patients with haematological malignancies [25]. On the other hand, another study conducted in India revealed a high infection rate in head and neck cancer patients with invasive fungal infection [18]. The use of broad-spectrum antibiotic may be one of the possible risk factors for candidemia infection in cancer patients [19].

Another major finding of this study is the susceptibility of cancer patients to antibiotics against particular pathogens. Of the total bacterial isolates found in 425 patients, only 19.84% were sensitive to all antibiotics, whereas 48% were MDR and 29% were XDR. Previous studies on the antimicrobial susceptibility pattern of cancer patients lack information on the prevalence and distribution of XDR bacteria, focusing mainly on MDR bacteria. On the other hand, our study provides a clear picture of the prevalence of both MDR and XDR bacteria, as well as the death rates associated with them [9, 12, 26]. Antibiotic susceptibility test results from our microbiology department show the highest drug resistance in gram negative bacterial isolates, this finding of antibiotic susceptibility for gram negative bacterial isolates contradicts earlier studies that showed high susceptibility to carbapenems and moderate susceptibility to cephalosporins, as well as β-lactam and β-lactamase inhibitors. This suggests an increase in resistance to last-resort antibiotics in other low- and middle-income countries, including India [11, 22, 28]. The high resistance to carbapenems and other classes of antibiotics may be due to the extensive and irrational use of broad-spectrum antibiotics for infection management in cancer patients. GPB showed susceptibility to the antibiotics tested. Only four cases of *vancomycin-resistant enterococci* have been reported, and some of the *MRSA* strains were also resistant to vancomycin.

This study investigated various factors that may be responsible for the development of antimicrobial resistance in cancer patients. We employed multinomial logistic regression to predict the risk factors for antimicrobial resistance, and identified a history of surgery, chemotherapy, exposure to antibiotics, hospitalization, severe neutropenia, and infection with gram-negative bacteria as possible risk factors for MDR bacteria. The same factors were responsible for the increased prevalence of XDR, except for gram-negative bacteria. Other studies have also noted that a WBC count less than 4000, nonfermenter gram-negative BSI, exposure to antibiotics within 3 months, chemotherapy, metastasis, and duration of hospital stay are possible risk factors contributing to the development of multidrug resistance in patients with gram-negative infections and in the intensive care unit [13, 22].

Bloodstream infections (BSIs) are among the leading causes of mortality and morbidity in cancer patients due to chemotherapy-induced neutropenia. This condition increases the vulnerability of these patients to infection. Other factors that increase the risk of BSIs include the use of indwelling catheters such as central venous catheters and PICCs, which can cause more hospital-acquired infections [17, 27]. The prevalence and the mortality of central line associated BSI were high in our study as compared to previous studies conducted in India [29]. The predominant pathogens causing bloodstream infections were *Klebsiella pneumoniae*, followed by *Klebsiella oxytoca* and Methicillin-resistant *Staphylococcus aureus*. The result differs with a study conducted in a tertiary cancer hospital in eastern India, where *E. coli* was the most commonly isolated bacteria in bloodstream infections, followed by *Klebsiella pneumoniae* [28]. In this study, the BSIs were the same in both haematological (49.27%) and solid tumour (50.73%) patients, which contradicts the findings of studies conducted in India and other countries [11, 18]. However, a study conducted in Mexico showed that patients with solid tumours had a higher incidence of BSIs than patients with haematological malignancies [30]. Monomicrobial and hospital-acquired infections were common among these patients, as most of them were admitted for chemotherapy. Almost 53% of patients with BSIs died compared to those with other types of infections, with an OR of 1.53 (CI 0.250–2.240), including bacteraemia, urinary tract infections (UTIs), and skin and soft tissue infections. The mortality rate due to BSI in our study was higher than that in previous studies conducted [13, 17, 27]. Therefore, proper management of high-risk patients for the prevention of bloodstream infections is paramount for reducing mortality and morbidity in these patients. This can be achieved by introducing an antimicrobial stewardship program in cancer institutes, following proper guidelines for the administration of antibiotics to febrile neutropenic patients and avoiding the irrational use of broad-spectrum antibiotics, thereby reducing the risk of developing MDR and XDR in bacteria.

Urinary tract infections (UTIs) are among the most neglected infections in comparison to bloodstream infections (BSIs) in cancer patients. In our study, UTI was the leading cause of infection, with a prevalence of 34%. This finding contradicts other studies that have demonstrated BSI as the major infection type [10, 31]. Additionally, 69.33% of the patients with UTIs had urinary catheters placed, which may be a possible reason for the higher incidence of UTIs. Our binomial logistic regression analysis for predictors of mortality showed high odds of mortality in cancer patients indwelled with Foley catheters, with an odds ratio of 4.13 (CI-1.720-9.925). Monomicrobial infections (87%), nosocomial infections (88.66%) and GNB (92.66%), including *K. pneumoniae* (22%) and *E. coli* (20.60%), were more common aetiology of UTIs. These findings contradict earlier studies conducted on cancer patients, which demonstrated a greater prevalence of *E. coli* than other Enterobacteriaceae pathogen groups [31, 32]. Another interesting observation was that UTIs caused by *Candida albicans* and *nonalbicans candida* accounted for 12%, indicating a shift in the epidemiology of UTIs from *E. coli* to yeast in recent years [33].

Skin and soft tissue infections due to surgeries for the management of cancer in the early stages have become common in cancer patients [34, 35]. In this study, such infections were the second most common after UTIs. Among patients diagnosed with skin and soft tissue infections, 43.89% underwent surgery during their stay at the hospital or had a prior history of surgery within three months of admission, suggesting a surgical site infection. Of these patients, 26% died within 30 days of the date of a positive culture. Moreover, patients with a history of surgery had an odds ratio of 1.139 for mortality, and patients who underwent surgery during their hospital stay had an odds ratio of 1.149. Patients who underwent surgery or who had a history of surgery within the last two to three months were at risk of surgical site infections. Since prophylactic antibiotics are prescribed to patients undergoing surgery, they are more susceptible to developing antimicrobial resistance, which increases the mortality rate in postsurgical patients and makes it difficult to manage infections with limited antibiotics. Proper preoperative and postoperative infection prevention strategies, along with appropriate antibiotic therapy, are crucial for preventing such infections.

Cancer patients are at greater risk of developing sepsis and septic shock, which can lead to mortality. A study conducted in Lebanon that compared the incidence of sepsis in patients with solid versus haematological cancer showed a similar risk of mortality in both types of patients, at approximately 47% [36]. In our study, patients with sepsis also exhibited 80% mortality, with an odds ratio (OR) of 6.79 (confidence interval [CI] = (1.23–37.68), *P* = 0.028. Some of the cancer patients had metastasis to other organs and had higher mortality, with an OR of 4.668 (CI = 1.341–16.25) (*P* = 0.15).

Patients with various invasive devices are at greater risk of acquiring healthcare-associated infections, such as central line-associated bloodstream infections, catheter-associated urinary tract infections, ventilator-associated pneumonia, and surgical site infections. These invasive devices were also associated with odds of death among cancer patients than among patients without any invasive devices. Central line-associated bloodstream infections led to 69.69% of deaths (OR 12.51, CI = 2.85–54.96), *P* < 0.001.

This is the first prospective study conducted in India to examine the risk factors associated with the development of antimicrobial resistance and mortality in cancer patients with a laboratory-confirmed diagnosis of infection. Based on the antibiotic susceptibility patterns, an antibiogram for predominant GNB and GPB was developed. This antibiogram will help physicians choose appropriate antibiotics, reduce the irrational use of antibiotics, decrease the risk of acquired antibiotic resistance, hospital-acquired infections, and the extra cost of medicines, and effectively manage infections, thereby reducing the length of hospital stay and mortality.

Although this study has several strengths, it also has some limitations that need to be considered. First, this study was conducted at a single centre, which means that further research should be performed at multiple centres to confirm the validity of the conclusions drawn from this study. Additionally, due to the small sample size, insufficient research has been conducted on the relationship between fungal infection impact and clinical outcomes, as well as on the risk factors for fungal resistance, survival analysis, and 30-day mortality.

## Conclusion

This study highlights a high prevalence of MDR and XDR infections in cancer patients, leading to increased morbidity and mortality. Chemotherapy, invasive devices, and high MASCC scores are possible risk factors for mortality. Factors like surgery, hospitalization, antibiotic use, and neutropenia likely contribute to AMR development. Strengthened infection control, regular surveillance, and utilizing hospital antibiograms are crucial to combat AMR and improve patient outcomes in cancer patients.

### Electronic supplementary material

Below is the link to the electronic supplementary material.

Supplementary Material 1

## Data Availability

The original data set is available with the first and corresponding authors and will be available upon request.

## Abbreviations

MDR: Multidrug-resistant
XDR: Extensively drug-resistant
GNB: Gram negative bacteria
GPB: Gram positive bacteria
AMR: Antimicrobial resistance
CLSI: Clinical Laboratory Standards Institute
NCI: National Cancer Institute
MASCC: Multinational Association for Supportive Care in Cancer
ATCC: American Type Culture Collection
MRSA: Methicillin-resistant *Staphylococcus aureus*
MSSA: Methicillin-sensitive *Staphylococcus aureus*
CoNs: Coagulase-negative *staphylococci *
BSIs: Blood stream infections
CVC: Central Venous catheter
PICC: Peripherally inserted central catheter
