# Supplementary material for: Investigation of antimicrobial susceptibility patterns, risk factors and their impact on mortality in cancer patients at a tertiary care cancer hospital- A prospective study
Source: Ann Clin Microbiol Antimicrob. 2024 Jun 26;23:59. doi: 10.1186/s12941-024-00703-5 (PMC11210011; doi:10.1186/s12941-024-00703-5)
Supplement: Supplementary file 1 — Supplementary Material 1 [file 12941_2024_703_MOESM1_ESM.docx]

**Supplementary file**

**
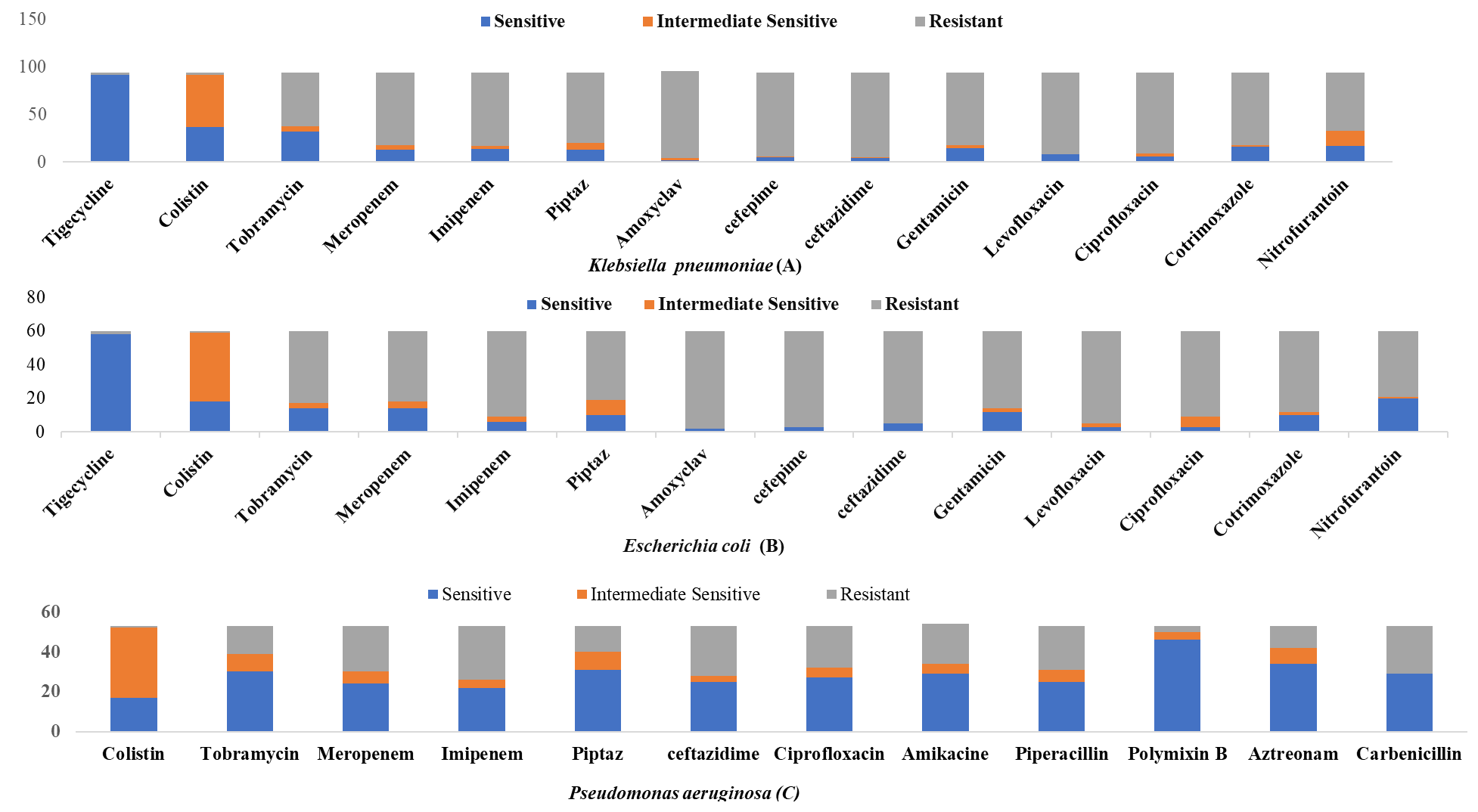
**

**Supplementary Figure 1(A-C).** **Antibiotic susceptibility pattern of Gram-negative bacteria**

Illustrates the antimicrobial susceptibility pattern of the predominant Gram-negative bacteria. The X-axis indicates the names of antibiotics that were tested against the specified bacteria. The blue coloration within each bar signifies the number of isolates that exhibit sensitivity to the particular antibiotics. However, the orange coloration reflects intermediate sensitivity, while the gray coloration indicates resistance to the antibiotics for each of the isolates. The Y-axis represents the total number of bacterial isolates

**
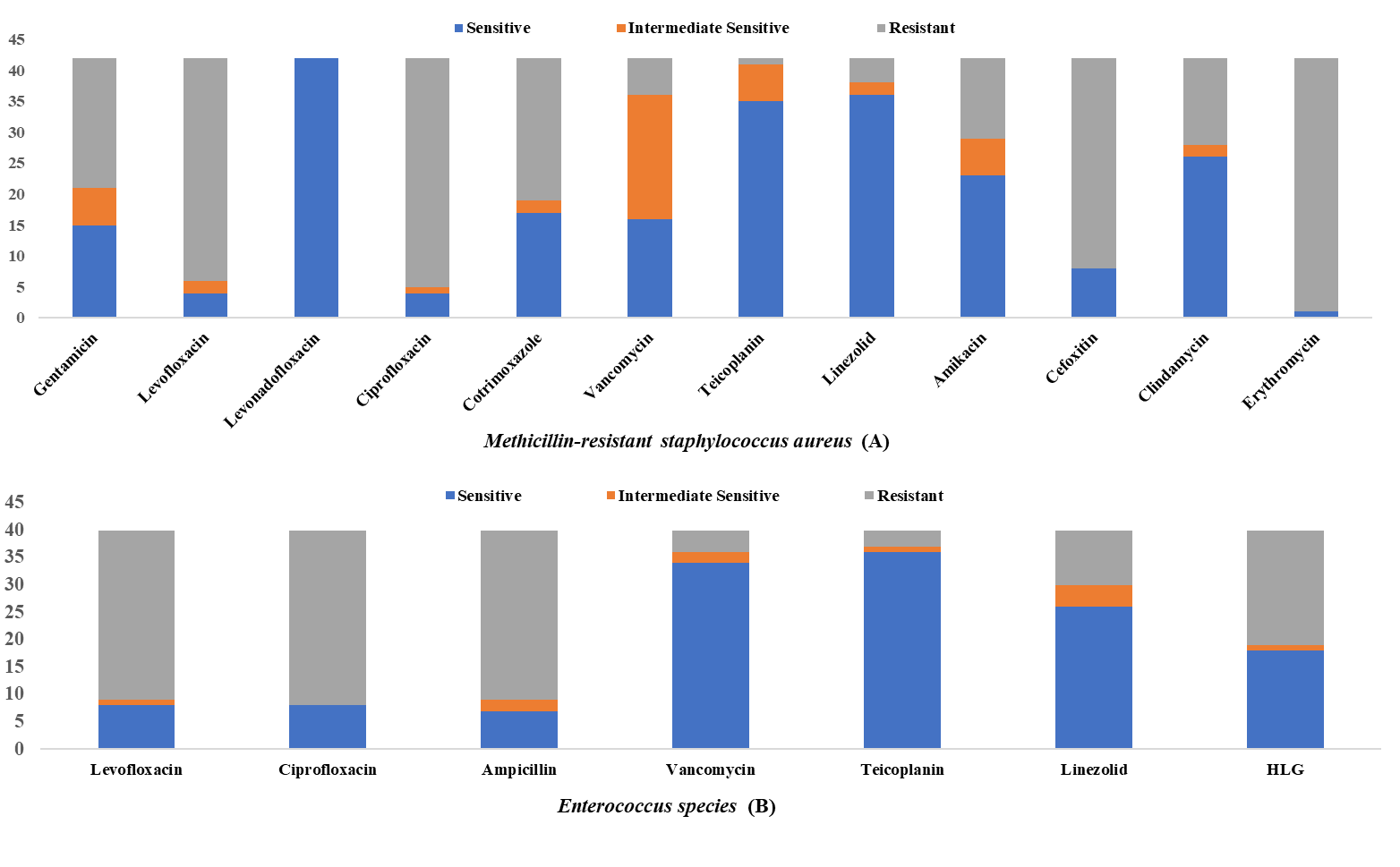
Supplementary Figure 2. Antibiotic susceptibility pattern of Gram-positive isolates**

Illustrates the antimicrobial susceptibility pattern of the predominant Gram-positive bacteria. The X-axis indicates the names of antibiotics that were tested against the specified bacteria. The blue coloration within each bar signifies the number of isolates that exhibit sensitivity to the particular antibiotics. However, the orange coloration reflects intermediate sensitivity, while the gray coloration indicates resistance to the antibiotics for each of the isolates. The Y-axis represents the total number of bacterial isolates.


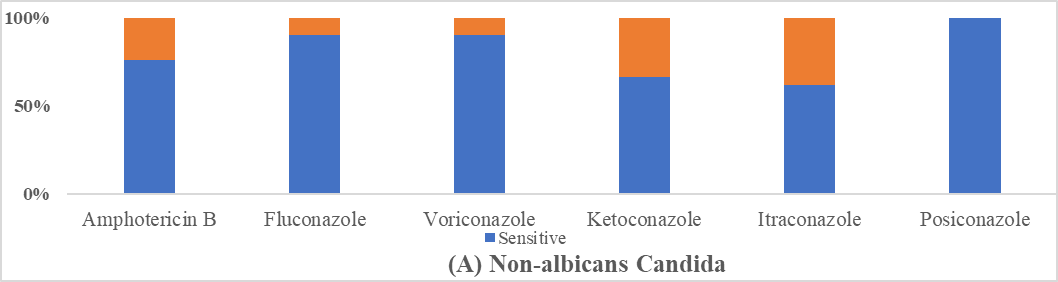

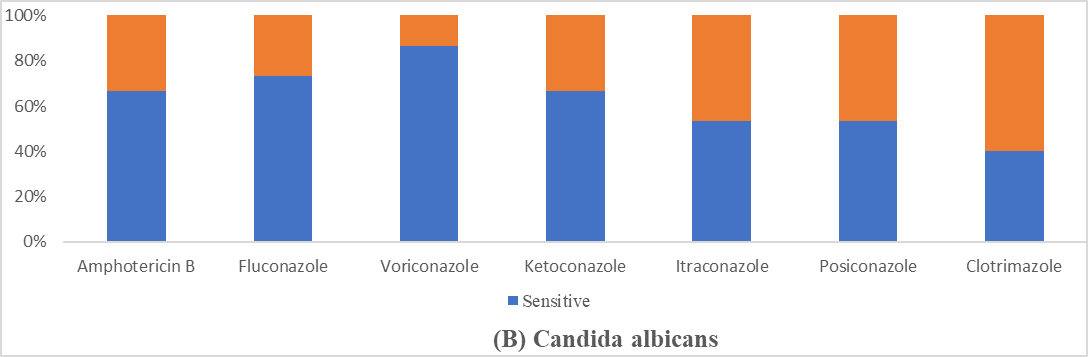


**Supplementary Figure 3:** Susceptibility pattern of fungal isolates


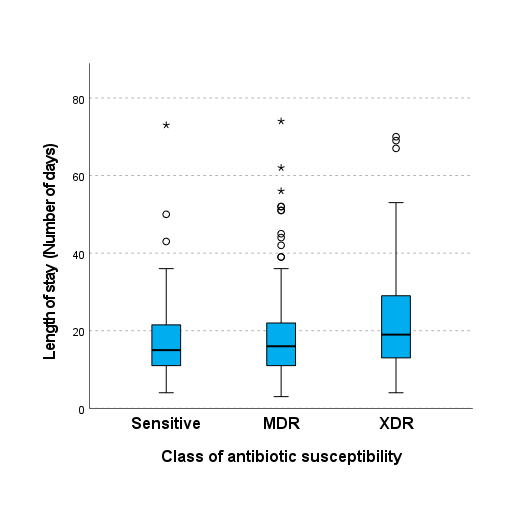


**Figure 4.** **Antibiotic susceptibility class and length of hospital stay**

**Supplementary Table 1** - Prevalence of Bacterial Isolates on the basis of Antibiotic susceptibility class and associated 30-day mortality in each cancer type.

| Diagnosis | Class of antibiotic susceptibility | 30-day mortality | Counts | % of Total | Cumulative % |
| --- | --- | --- | --- | --- | --- |
| Acute leukaemia | Sensitive | Dead | 1 | 0.2 % | 0.2 % |
|  |  | Alive | 9 | 2.1 % | 2.4 % |
|  | MDR | Dead | 8 | 1.9 % | 4.2 % |
|  |  | Alive | 11 | 2.6 % | 6.8 % |
|  | XDR | Dead | 11 | 2.6 % | 9.4 % |
|  |  | Alive | 1 | 0.2 % | 9.6 % |
| Astrocytoma | Sensitive | Dead | 0 | 0.0 % | 9.6 % |
|  |  | Alive | 1 | 0.2 % | 9.9 % |
|  | MDR | Dead | 0 | 0.0 % | 9.9 % |
|  |  | Alive | 0 | 0.0 % | 9.9 % |
|  | XDR | Dead | 0 | 0.0 % | 9.9 % |
|  |  | Alive | 0 | 0.0 % | 9.9 % |
| Breast | Sensitive | Dead | 0 | 0.0 % | 9.9 % |
|  |  | Alive | 15 | 3.5 % | 13.4 % |
|  | MDR | Dead | 1 | 0.2 % | 13.6 % |
|  |  | Alive | 13 | 3.1 % | 16.7 % |
|  | XDR | Dead | 8 | 1.9 % | 18.6 % |
|  |  | Alive | 3 | 0.7 % | 19.3 % |
| Burkitts lymphoma | Sensitive | Dead | 0 | 0.0 % | 19.3 % |
|  |  | Alive | 1 | 0.2 % | 19.5 % |
|  | MDR | Dead | 0 | 0.0 % | 19.5 % |
|  |  | Alive | 1 | 0.2 % | 19.8 % |
|  | XDR | Dead | 0 | 0.0 % | 19.8 % |
|  |  | Alive | 0 | 0.0 % | 19.8 % |
| Cervix | Sensitive | Dead | 1 | 0.2 % | 20.0 % |
|  |  | Alive | 9 | 2.1 % | 22.1 % |
|  | MDR | Dead | 4 | 0.9 % | 23.1 % |
|  |  | Alive | 25 | 5.9 % | 28.9 % |
|  | XDR | Dead | 11 | 2.6 % | 31.5 % |
|  |  | Alive | 4 | 0.9 % | 32.5 % |
| Chronic leukemia | Sensitive | Dead | 1 | 0.2 % | 32.7 % |
|  |  | Alive | 1 | 0.2 % | 32.9 % |
|  | MDR | Dead | 3 | 0.7 % | 33.6 % |
|  |  | Alive | 3 | 0.7 % | 34.4 % |
|  | XDR | Dead | 1 | 0.2 % | 34.6 % |
|  |  | Alive | 2 | 0.5 % | 35.1 % |
| Colorectal cancer | Sensitive | Dead | 2 | 0.5 % | 35.5 % |
|  |  | Alive | 4 | 0.9 % | 36.5 % |
|  | MDR | Dead | 2 | 0.5 % | 36.9 % |
|  |  | Alive | 10 | 2.4 % | 39.3 % |
|  | XDR | Dead | 2 | 0.5 % | 39.8 % |
|  |  | Alive | 13 | 3.1 % | 42.8 % |
| Common bile duct | Sensitive | Dead | 0 | 0.0 % | 42.8 % |
|  |  | Alive | 0 | 0.0 % | 42.8 % |
|  | MDR | Dead | 0 | 0.0 % | 42.8 % |
|  |  | Alive | 1 | 0.2 % | 43.1 % |
|  | XDR | Dead | 0 | 0.0 % | 43.1 % |
|  |  | Alive | 1 | 0.2 % | 43.3 % |
| Ear | Sensitive | Dead | 0 | 0.0 % | 43.3 % |
|  |  | Alive | 1 | 0.2 % | 43.5 % |
|  | MDR | Dead | 0 | 0.0 % | 43.5 % |
|  |  | Alive | 0 | 0.0 % | 43.5 % |
|  | XDR | Dead | 0 | 0.0 % | 43.5 % |
|  |  | Alive | 0 | 0.0 % | 43.5 % |
| Ewings sarcoma | Sensitive | Dead | 0 | 0.0 % | 43.5 % |
|  |  | Alive | 1 | 0.2 % | 43.8 % |
|  | MDR | Dead | 3 | 0.7 % | 44.5 % |
|  |  | Alive | 2 | 0.5 % | 44.9 % |
|  | XDR | Dead | 0 | 0.0 % | 44.9 % |
|  |  | Alive | 2 | 0.5 % | 45.4 % |
| Giant cell Tumor | Sensitive | Dead | 0 | 0.0 % | 45.4 % |
|  |  | Alive | 0 | 0.0 % | 45.4 % |
|  | MDR | Dead | 0 | 0.0 % | 45.4 % |
|  |  | Alive | 0 | 0.0 % | 45.4 % |
|  | XDR | Dead | 1 | 0.2 % | 45.6 % |
|  |  | Alive | 1 | 0.2 % | 45.9 % |
| Gall bladder | Sensitive | Dead | 1 | 0.2 % | 46.1 % |
|  |  | Alive | 6 | 1.4 % | 47.5 % |
|  | MDR | Dead | 6 | 1.4 % | 48.9 % |
|  |  | Alive | 11 | 2.6 % | 51.5 % |
|  | XDR | Dead | 10 | 2.4 % | 53.9 % |
|  |  | Alive | 5 | 1.2 % | 55.1 % |
| Glioblastoma | Sensitive | Dead | 1 | 0.2 % | 55.3 % |
|  |  | Alive | 0 | 0.0 % | 55.3 % |
|  | MDR | Dead | 1 | 0.2 % | 55.5 % |
|  |  | Alive | 0 | 0.0 % | 55.5 % |
|  | XDR | Dead | 1 | 0.2 % | 55.8 % |
|  |  | Alive | 0 | 0.0 % | 55.8 % |
| Head and neck cancer | Sensitive | Dead | 2 | 0.5 % | 56.2 % |
|  |  | Alive | 14 | 3.3 % | 59.5 % |
|  | MDR | Dead | 8 | 1.9 % | 61.4 % |
|  |  | Alive | 18 | 4.2 % | 65.6 % |
|  | XDR | Dead | 7 | 1.6 % | 67.3 % |
|  |  | Alive | 6 | 1.4 % | 68.7 % |
| Hodgkings | Sensitive | Dead | 0 | 0.0 % | 68.7 % |
|  |  | Alive | 0 | 0.0 % | 68.7 % |
|  | MDR | Dead | 0 | 0.0 % | 68.7 % |
|  |  | Alive | 0 | 0.0 % | 68.7 % |
|  | XDR | Dead | 1 | 0.2 % | 68.9 % |
|  |  | Alive | 0 | 0.0 % | 68.9 % |
| Kidney | Sensitive | Dead | 0 | 0.0 % | 68.9 % |
|  |  | Alive | 0 | 0.0 % | 68.9 % |
|  | MDR | Dead | 2 | 0.5 % | 69.4 % |
|  |  | Alive | 0 | 0.0 % | 69.4 % |
|  | XDR | Dead | 0 | 0.0 % | 69.4 % |
|  |  | Alive | 0 | 0.0 % | 69.4 % |
| Liver | Sensitive | Dead | 0 | 0.0 % | 69.4 % |
|  |  | Alive | 0 | 0.0 % | 69.4 % |
|  | MDR | Dead | 1 | 0.2 % | 69.6 % |
|  |  | Alive | 5 | 1.2 % | 70.8 % |
|  | XDR | Dead | 0 | 0.0 % | 70.8 % |
|  |  | Alive | 0 | 0.0 % | 70.8 % |
| Lower limb | Sensitive | Dead | 0 | 0.0 % | 70.8 % |
|  |  | Alive | 4 | 0.9 % | 71.8 % |
|  | MDR | Dead | 1 | 0.2 % | 72.0 % |
|  |  | Alive | 2 | 0.5 % | 72.5 % |
|  | XDR | Dead | 3 | 0.7 % | 73.2 % |
|  |  | Alive | 3 | 0.7 % | 73.9 % |
| Lung | Sensitive | Dead | 1 | 0.2 % | 74.1 % |
|  |  | Alive | 3 | 0.7 % | 74.8 % |
|  | MDR | Dead | 4 | 0.9 % | 75.8 % |
|  |  | Alive | 6 | 1.4 % | 77.2 % |
|  | XDR | Dead | 2 | 0.5 % | 77.6 % |
|  |  | Alive | 2 | 0.5 % | 78.1 % |
| Meningioma | Sensitive | Dead | 0 | 0.0 % | 78.1 % |
|  |  | Alive | 0 | 0.0 % | 78.1 % |
|  | MDR | Dead | 0 | 0.0 % | 78.1 % |
|  |  | Alive | 1 | 0.2 % | 78.4 % |
|  | XDR | Dead | 0 | 0.0 % | 78.4 % |
|  |  | Alive | 0 | 0.0 % | 78.4 % |
| Multiple myeloma | Sensitive | Dead | 0 | 0.0 % | 78.4 % |
|  |  | Alive | 1 | 0.2 % | 78.6 % |
|  | MDR | Dead | 1 | 0.2 % | 78.8 % |
|  |  | Alive | 5 | 1.2 % | 80.0 % |
|  | XDR | Dead | 1 | 0.2 % | 80.2 % |
|  |  | Alive | 1 | 0.2 % | 80.5 % |
| Non-hodgkin’s lymphoma | Sensitive | Dead | 1 | 0.2 % | 80.7 % |
|  |  | Alive | 0 | 0.0 % | 80.7 % |
|  | MDR | Dead | 2 | 0.5 % | 81.2 % |
|  |  | Alive | 4 | 0.9 % | 82.1 % |
|  | XDR | Dead | 1 | 0.2 % | 82.4 % |
|  |  | Alive | 1 | 0.2 % | 82.6 % |
| Nose | Sensitive | Dead | 0 | 0.0 % | 82.6 % |
|  |  | Alive | 0 | 0.0 % | 82.6 % |
|  | MDR | Dead | 0 | 0.0 % | 82.6 % |
|  |  | Alive | 1 | 0.2 % | 82.8 % |
|  | XDR | Dead | 0 | 0.0 % | 82.8 % |
|  |  | Alive | 0 | 0.0 % | 82.8 % |
| Ovary | Sensitive | Dead | 0 | 0.0 % | 82.8 % |
|  |  | Alive | 1 | 0.2 % | 83.1 % |
|  | MDR | Dead | 0 | 0.0 % | 83.1 % |
|  |  | Alive | 6 | 1.4 % | 84.5 % |
|  | XDR | Dead | 1 | 0.2 % | 84.7 % |
|  |  | Alive | 3 | 0.7 % | 85.4 % |
| Pancreas | Sensitive | Dead | 0 | 0.0 % | 85.4 % |
|  |  | Alive | 0 | 0.0 % | 85.4 % |
|  | MDR | Dead | 0 | 0.0 % | 85.4 % |
|  |  | Alive | 3 | 0.7 % | 86.1 % |
|  | XDR | Dead | 0 | 0.0 % | 86.1 % |
|  |  | Alive | 2 | 0.5 % | 86.6 % |
| Penis | Sensitive | Dead | 0 | 0.0 % | 86.6 % |
|  |  | Alive | 2 | 0.5 % | 87.1 % |
|  | MDR | Dead | 0 | 0.0 % | 87.1 % |
|  |  | Alive | 1 | 0.2 % | 87.3 % |
|  | XDR | Dead | 2 | 0.5 % | 87.8 % |
|  |  | Alive | 2 | 0.5 % | 88.2 % |
| Periampullary | Sensitive | Dead | 0 | 0.0 % | 88.2 % |
|  |  | Alive | 0 | 0.0 % | 88.2 % |
|  | MDR | Dead | 0 | 0.0 % | 88.2 % |
|  |  | Alive | 1 | 0.2 % | 88.5 % |
|  | XDR | Dead | 0 | 0.0 % | 88.5 % |
|  |  | Alive | 1 | 0.2 % | 88.7 % |
| Pleomorphic rhabdomyosarcoma | Sensitive | Dead | 1 | 0.2 % | 88.9 % |
|  |  | Alive | 0 | 0.0 % | 88.9 % |
|  | MDR | Dead | 0 | 0.0 % | 88.9 % |
|  |  | Alive | 0 | 0.0 % | 88.9 % |
|  | XDR | Dead | 0 | 0.0 % | 88.9 % |
|  |  | Alive | 0 | 0.0 % | 88.9 % |
| Prostate | Sensitive | Dead | 0 | 0.0 % | 88.9 % |
|  |  | Alive | 0 | 0.0 % | 88.9 % |
|  | MDR | Dead | 1 | 0.2 % | 89.2 % |
|  |  | Alive | 4 | 0.9 % | 90.1 % |
|  | XDR | Dead | 2 | 0.5 % | 90.6 % |
|  |  | Alive | 0 | 0.0 % | 90.6 % |
| Stomach | Sensitive | Dead | 1 | 0.2 % | 90.8 % |
|  |  | Alive | 3 | 0.7 % | 91.5 % |
|  | MDR | Dead | 1 | 0.2 % | 91.8 % |
|  |  | Alive | 1 | 0.2 % | 92.0 % |
|  | XDR | Dead | 0 | 0.0 % | 92.0 % |
|  |  | Alive | 1 | 0.2 % | 92.2 % |
| Testies | Sensitive | Dead | 0 | 0.0 % | 92.2 % |
|  |  | Alive | 0 | 0.0 % | 92.2 % |
|  | MDR | Dead | 1 | 0.2 % | 92.5 % |
|  |  | Alive | 1 | 0.2 % | 92.7 % |
|  | XDR | Dead | 0 | 0.0 % | 92.7 % |
|  |  | Alive | 0 | 0.0 % | 92.7 % |
| Thyroid | Sensitive | Dead | 0 | 0.0 % | 92.7 % |
|  |  | Alive | 0 | 0.0 % | 92.7 % |
|  | MDR | Dead | 1 | 0.2 % | 92.9 % |
|  |  | Alive | 2 | 0.5 % | 93.4 % |
|  | XDR | Dead | 1 | 0.2 % | 93.6 % |
|  |  | Alive | 0 | 0.0 % | 93.6 % |
| Upper limb | Sensitive | Dead | 0 | 0.0 % | 93.6 % |
|  |  | Alive | 2 | 0.5 % | 94.1 % |
|  | MDR | Dead | 0 | 0.0 % | 94.1 % |
|  |  | Alive | 2 | 0.5 % | 94.6 % |
|  | XDR | Dead | 0 | 0.0 % | 94.6 % |
|  |  | Alive | 0 | 0.0 % | 94.6 % |
| Urinary bladder | Sensitive | Dead | 0 | 0.0 % | 94.6 % |
|  |  | Alive | 4 | 0.9 % | 95.5 % |
|  | MDR | Dead | 2 | 0.5 % | 96.0 % |
|  |  | Alive | 4 | 0.9 % | 96.9 % |
|  | XDR | Dead | 1 | 0.2 % | 97.2 % |
|  |  | Alive | 1 | 0.2 % | 97.4 % |
| Uterus | Sensitive | Dead | 0 | 0.0 % | 97.4 % |
|  |  | Alive | 3 | 0.7 % | 98.1 % |
|  | MDR | Dead | 0 | 0.0 % | 98.1 % |
|  |  | Alive | 3 | 0.7 % | 98.8 % |
|  | XDR | Dead | 1 | 0.2 % | 99.1 % |
|  |  | Alive | 1 | 0.2 % | 99.3 % |
| Vagina | Sensitive | Dead | 0 | 0.0 % | 99.3 % |
|  |  | Alive | 1 | 0.2 % | 99.5 % |
|  | MDR | Dead | 0 | 0.0 % | 99.5 % |
|  |  | Alive | 0 | 0.0 % | 99.5 % |
|  | XDR | Dead | 0 | 0.0 % | 99.5 % |
|  |  | Alive | 0 | 0.0 % | 99.5 % |
| Vulva | Sensitive | Dead | 0 | 0.0 % | 99.5 % |
|  |  | Alive | 1 | 0.2 % | 99.8 % |
|  | MDR | Dead | 0 | 0.0 % | 99.8 % |
|  |  | Alive | 1 | 0.2 % | 100.0 % |
|  | XDR | Dead | 0 | 0.0 % | 100.0 % |
|  |  | Alive | 0 | 0.0 % | 100.0 % |

**Supplementary Table 2** Multinomial logistic regression analysis for prediction of antimicrobial resistance in cancer patients

| **Parameter Estimates** | | | | | | | | | |
| --- | --- | --- | --- | --- | --- | --- | --- | --- | --- |
| Class of Resistance^a^ | | B | Std. Error | Wald | df | Sig. | Exp(B) | 95% Confidence Interval for Exp(B) | |
|  |  |  |  |  |  |  |  | Lower Bound | Upper Bound |
| MDR | Intercept | 51.544 | 189.323 | .001 | 1 | .974 |  |  |  |
|  | Diagnosis Type | .005 | .323 | .000 | 1 | .988 | 1.005 | .534 | 1.891 |
|  | Type of Cancer | -7.518 | 349.129 | .000 | 1 | .983 | .001 | 3.595 | 8.201 |
|  | Stage | .123 | .346 | .126 | 1 | .723 | 1.131 | .574 | 2.229 |
|  | Histological Classification | .866 | .784 | 1.219 | 1 | .270 | 2.377 | .511 | 11.050 |
|  | Cardiovascular Comorbidity | .575 | .517 | 1.237 | 1 | .266 | 1.777 | .645 | 4.898 |
|  | Pneumonia | -11.129 | 72.311 | .024 | 1 | .878 | 1.468 | 4.127 | 5.256 |
|  | Metastasis | -.089 | .841 | .011 | 1 | .916 | .915 | .176 | 4.755 |
|  | Jaundice | -1.963 | 1.348 | 2.119 | 1 | .145 | .140 | .010 | 1.974 |
|  | Sepsis | -1.077 | 1.289 | .698 | 1 | .403 | .340 | .027 | 4.262 |
|  | Anemia | -.041 | .804 | .003 | 1 | .959 | .960 | .199 | 4.637 |
|  | Hypothyroidisam | -.844 | .614 | 1.891 | 1 | .169 | .430 | .129 | 1.432 |
|  | Prior H/O surgery | 1.005 | .365 | 7.578 | 1 | .006 | 2.731 | 1.336 | 5.586 |
|  | H/O Radiation | -1.140 | .542 | 4.425 | 1 | .035 | .320 | .111 | .925 |
|  | H/O Chemo | 1.600 | .425 | 14.138 | 1 | .000 | 4.952 | 2.151 | 11.400 |
|  | Treatment Given | -.048 | .146 | .108 | 1 | .743 | .953 | .717 | 1.268 |
|  | Tracheostomy | .723 | 1.095 | .436 | 1 | .509 | 2.060 | .241 | 17.605 |
|  | IV Canulla | 0^b^ | . | . | 0 | . | . | . | . |
|  | Foleys catheter | -.040 | .385 | .011 | 1 | .918 | .961 | .452 | 2.044 |
|  | RT | -.228 | .419 | .297 | 1 | .586 | .796 | .351 | 1.808 |
|  | CVC | -.369 | .839 | .194 | 1 | .660 | .691 | .134 | 3.575 |
|  | NCI Category | .540 | .536 | 1.018 | 1 | .313 | 1.717 | .601 | 4.904 |
|  | MASCC | -.161 | .396 | .165 | 1 | .684 | .851 | .391 | 1.851 |
|  | Type of Sample | -.055 | .864 | .004 | 1 | .950 | .947 | .174 | 5.152 |
|  | Pathogen identified | -.335 | .075 | 20.007 | 1 | .000 | .716 | .618 | .829 |
|  | Polymicrobial/Monomicrobial | -.969 | .465 | 4.343 | 1 | .037 | .379 | .152 | .944 |
|  | Type of Bacteria | 3.155 | .933 | 11.443 | 1 | .001 | 23.446 | 3.769 | 145.845 |
|  | Type of infection | -.659 | .386 | 2.910 | 1 | .088 | .517 | .243 | 1.103 |
|  | Use of Antibiotics | -.497 | .432 | 1.324 | 1 | .001 | 2.460 | 1.741 | 6.481 |
|  | History of Hospitalisation | 1.075 | .507 | 4.489 | 1 | .034 | 2.930 | 1.084 | 7.922 |
|  | Recent | .681 | .684 | .991 | 1 | .319 | 1.975 | .517 | 7.544 |
|  | Progression | 0^b^ | . | . | 0 | . | . | . | . |
|  | Solid Tumor | 0^b^ | . | . | 0 | . | . | . | . |
|  | Heamatological | 0^b^ | . | . | 0 | . | . | . | . |
|  | Stage-II | .084 | .513 | .027 | 1 | .870 | 1.088 | .398 | 2.973 |
|  | Stage-III | .232 | .639 | .131 | 1 | .717 | 1.261 | .361 | 4.407 |
|  | Stage-IV | .204 | .966 | .045 | 1 | .833 | 1.227 | .185 | 8.154 |
|  | Carcinoma | -4.480 | 574.684 | .000 | 1 | .994 | .011 | .000 | .^c^ |
|  | Leukemia | -11.206 | 527.511 | .000 | 1 | .983 | 1.36 | .000 | .^c^ |
|  | HTN | -2.248 | 1.542 | 2.125 | 1 | .145 | .106 | .005 | 2.170 |
|  | HTN+DM | -2.033 | 1.136 | 3.201 | 1 | .074 | .131 | .014 | 1.214 |
|  | DM | 0^b^ | . | . | 0 | . | . | . | . |
|  | NCI-III | -.077 | 1.933 | .002 | 1 | .968 | .926 | .021 | 40.872 |
|  | NCI-IV | .862 | 2.056 | .176 | 1 | .009 | 1.247 | 1.042 | 1.426 |
|  | Neutrophillia | 1.717 | 2.616 | .431 | 1 | .512 | 5.567 | .033 | 937.926 |
|  | Pus | 1.075 | 4.353 | .061 | 1 | .805 | 2.930 | .001 | 14856.720 |
|  | Urine | .657 | 3.526 | .035 | 1 | .852 | 1.929 | .002 | 1933.925 |
|  | Blood | 1.979 | 2.736 | .523 | 1 | .469 | 7.239 | .034 | 1544.927 |
|  | Sputum | .990 | 2.019 | .241 | 1 | .624 | 2.692 | .051 | 140.711 |
|  | Chemotherapy | -.415 | .884 | .221 | 1 | .638 | .660 | .117 | 3.730 |
|  | Surgery | .852 | .776 | 1.205 | 1 | .272 | 2.344 | .512 | 10.721 |
|  | Female | -.270 | .354 | .581 | 1 | .446 | .763 | .381 | 1.528 |
|  | Male | 0^b^ | . | . | 0 | . | . | . | . |
| XDR | Intercept | -3.306 | 148.141 | .000 | 1 | .982 |  |  |  |
|  | Diagnosis Type | -.069 | .439 | .025 | 1 | .875 | .933 | .394 | 2.208 |
|  | Type of Cancer | 2.930 | 397.148 | .000 | 1 | .994 | 18.722 | .000 | .^c^ |
|  | Stage | .864 | .428 | 4.085 | 1 | .043 | 2.374 | 1.027 | 5.488 |
|  | Histological Classification | 1.633 | .859 | 3.617 | 1 | .057 | 5.119 | .951 | 27.545 |
|  | Cardiovascular morbidity | 1.381 | .676 | 4.173 | 1 | .041 | 3.978 | 1.057 | 14.961 |
|  | Pneumonia | -11.202 | 72.316 | .024 | 1 | .877 | 1.365 | 3.802 | 4.900 |
|  | Metastasis | -1.583 | .909 | 3.033 | 1 | .082 | .205 | .035 | 1.220 |
|  | Jaundice | -2.438 | 1.457 | 2.798 | 1 | .094 | .087 | .005 | 1.520 |
|  | Sepsis | -2.121 | 1.349 | 2.472 | 1 | .116 | .120 | .009 | 1.687 |
|  | Anemia | .330 | 1.150 | .083 | 1 | .774 | 1.391 | .146 | 13.256 |
|  | Hypothyroidisam | -.431 | .684 | .398 | 1 | .528 | .650 | .170 | 2.481 |
|  | Prior H/O surgery | .652 | .439 | 2.209 | 1 | .040 | 1.920 | 1.562 | 4.535 |
|  | H/O Radiotherapy | -.712 | .648 | 1.205 | 1 | .272 | .491 | .138 | 1.749 |
|  | H/O Chemotherapy | 1.346 | .487 | 7.650 | 1 | .006 | 3.841 | 1.480 | 9.968 |
|  | Treatment Given | .080 | .174 | .210 | 1 | .647 | 1.083 | .770 | 1.523 |
|  | Tracheostomy | 1.494 | 1.330 | 1.262 | 1 | .261 | 4.455 | .329 | 60.387 |
|  | IV Canulla | 0^b^ | . | . | 0 | . | . | . | . |
|  | Foleys catheter | -.440 | .476 | .857 | 1 | .355 | .644 | .253 | 1.636 |
|  | RT | -.574 | .488 | 1.381 | 1 | .240 | .563 | .216 | 1.467 |
|  | CVC | -.748 | .896 | .697 | 1 | .404 | .473 | .082 | 2.742 |
|  | NCI Category | .612 | .788 | .603 | 1 | .437 | 1.844 | .394 | 8.640 |
|  | MASCC | -1.071 | .472 | 5.157 | 1 | .023 | .343 | .136 | .864 |
|  | Type of Sample | -.380 | 1.097 | .120 | 1 | .729 | .684 | .080 | 5.869 |
|  | Pathogen identified | -.402 | .088 | 20.887 | 1 | .000 | .669 | .563 | .795 |
|  | Polymicrobial/Monomicrobial | .143 | .512 | .078 | 1 | .780 | 1.154 | .423 | 3.148 |
|  | Type of Bacteria | -.326 | 1.503 | .047 | 1 | .828 | .722 | .038 | 13.732 |
|  | Type of infection | -.994 | .465 | 4.564 | 1 | .033 | .370 | .149 | .921 |
|  | Use of Antibiotics | -.303 | .496 | .372 | 1 | .021 | 3.432 | 1.650 | 10.842 |
|  | History of Hospitalisation | 1.355 | .577 | 5.520 | 1 | .019 | 3.877 | 1.252 | 12.004 |
|  | Recent | .596 | .872 | .467 | 1 | .494 | 1.815 | .328 | 10.034 |
|  | Progression | 0^b^ | . | . | 0 | . | . | . | . |
|  | Solid Tumor | 0^b^ | . | . | 0 | . | . | . | . |
|  | Hematological | 0^b^ | . | . | 0 | . | . | . | . |
|  | Stage-II | -.022 | .705 | .001 | 1 | .975 | .978 | .246 | 3.897 |
|  | Stage-III | -.240 | .739 | .105 | 1 | .746 | .787 | .185 | 3.351 |
|  | Stage-IV | .500 | 1.107 | .204 | 1 | .651 | 1.649 | .188 | 14.450 |
|  | Carcinoma | 6.563 | 397.149 | .000 | 1 | .987 | 708.187 | .000 | .^c^ |
|  | Leukemia | 13.406 | .000 | . | 1 | .091 | 2.351 | 1.238 | 3.785 |
|  | HTN | -4.012 | 2.012 | 3.976 | 1 | .046 | .018 | .000 | .934 |
|  | HTN+DM | -3.564 | 1.479 | 5.809 | 1 | .016 | .028 | .002 | .514 |
|  | DM | 0^b^ | . | . | 0 | . | . | . | . |
|  | NCI-III | -.559 | 2.735 | .042 | 1 | .838 | .572 | .003 | 121.596 |
|  | NCI-IV | .661 | 3.054 | .047 | 1 | .001 | 1.346 | 1.121 | 1.616 |
|  | Neutrophillia | 1.598 | 3.843 | .173 | 1 | .678 | 4.941 | .003 | 9217.130 |
|  | Pus | 1.053 | 5.394 | .038 | 1 | .845 | 2.866 | 7.347 | 15.683 |
|  | Urine | 1.001 | 4.340 | .053 | 1 | .818 | 2.720 | .001 | 13441.982 |
|  | Blood | 1.081 | 3.326 | .106 | 1 | .745 | 2.948 | .004 | 1996.977 |
|  | Sputum | .441 | 2.413 | .033 | 1 | .855 | 1.554 | .014 | 176.065 |
|  | Chemotherapy | -.969 | 1.059 | .837 | 1 | .360 | .379 | .048 | 3.025 |
|  | Surgery | .330 | .934 | .125 | 1 | .724 | 1.391 | .223 | 8.675 |
|  | Female | -.636 | .438 | 2.111 | 1 | .146 | .529 | .224 | 1.249 |
|  | Male | 0^b^ | . | . | 0 | . | . | . | . |
| a. The reference category is: Sensitive. | | | | | | | | | |
| b. This parameter is set to zero because it is redundant. | | | | | | | | | |
| c. Floating point overflow occurred while computing this statistic. Its value is therefore set to system missing. | | | | | | | | | |

**Supplementary Table 3** Bivariate logistic regression analysis for prediction of risk factors for 30-day Mortality

| **Factor** | **30-Day survival (n=300)** | **30- Day mortality(n=140)** | **OR**  **(CI-95%)** | **p-value*** |
| --- | --- | --- | --- | --- |
| Recurrence  Remission | 41(13.7)  34(11.3) | 25(17.9)  1(0.7) | 27.65(2.00-38.75)  0.29(.06-14.61) | 0.013  0.534 |
| Solid Tumour | 264(88.0) | 111(79.3) | 0.519(0.31-0.87) | 0.013 |
| Stage-I  Stages-II  Stages-III  Stages-IV | 26(8.7)  114(38.0)  100(33.3)  22(7.3) | 1(0.7)  43(30.7)  46(32.9)  20(14.3) | 0.11(0.01-1.22)  0.05(0.01-0.63)  0.03(0.02-0.48)  0.84(.02-108.37) | 0.071  0.020  0.013  0.001 |
| HTN  DM  HTN+DM  Jaundice  Sepsis  Anaemia  Hypothyroidism  Metastasis | 40(13.3)  27(9.0)  26(8.7)  8(2.66)  4(1.33)  11(3.66)  34(11.33)   31(7.04) | 17(25.0)  10(7.1)  18(12.85)  1(0.71)  16(11.42)  3(2.14)  12(8.57)  19(13.57) | 0.504(0.095-2.676)  0.125(0.029-.549)  0.436(0.143-1.34)  0.034(0.002-0.598)  6.790(1.23-37.68)  0.499(0.070-3.559)  0.556(0.163-1.894)  4.668(1.341-16.25) | 0.421  0.145  0.006  0.021  0.028  0.488  0.348  0.150 |
| History of Surgery  History of Radiation  History of Chemotherapy | 88(29.3)  49(16.0)  109(36.00) | 41(29.28)  26(18.57)  55(39.28) | 1.275(0.581-2.792)  2.038(0.710-5.855)  1.439(0.604-3.425) | 0.54  0.18  0.41 |
| Chemotherapy  Radiotherapy  Surgery | 98(32.7)  16(5.3)  93(31.0) | 50(35.7)  8(5.7)  23(16.4) | 3.546(1.06-11.805)  2.400(.425-13.540)  1.449(.253-8.304) | 0.039  0.321  0.67 |
| Foley Catheter  RT  CVC  Tracheostomy  ET Tube | 162(36.81)  69(15.68)  10 (2.72)   12(2.72)  07(1.59) | 112(25.45)  74(16.81)  23(5.22)  08(2.00)  06(1.40) | 4.13(1.720-9.925)  2.74(1.188-6.335)  12.51(2.85-54.96)  17.23(0.28-104.84)  2.02(.057-72.178) | 0.002  0.018  <0.001  0.173  0.699 |
| NCI Category-III  NCI Category-IV  Neutrophilia | 8(2.7)  21(7.0)  141(47.0) | 3(2.1)  20(14.3)  88(62.85) | 1.096(.084-14.231)  1.395(.082-1.890)  1.265(0.111-0.631) | 0.045  0.002  0.003 |
| High Risk (<21) | 32(31.3) | 142(68.7) | 2.493(1.083-5.738) | 0.032* |
| *Klebsiella Pneumoniae* | 57(17.3) | 37(27.61) | 1.40(0.46-.20) | 0.553 |
| *E.Coli* | 41(12.0) | 19(14.17) | 1.106(0.314-3.896) | 0.875 |
| *Klebsiella oxytoca* | 37(11.0) | 21(15.67) | 0.430(0.094-1.969) | 0.277 |
| *Pseudomona aeruginosa* | 14(4.7) | 19(14.17) | 1.532(0.315-7.447) | 0.597 |
| *Klebsiella Spp.* | 24(8.0) | 14(10.44) | 0.883(0.137-5.701) | 0.896 |
| *Acinetobacter baumannii* | 12(4.0 | 12(8.9) | 1.25(.155-10.082) | 0.835 |
| *Pseudomonas spp.* | 14(4.7) | 10(7.44) | 1.55(.09-26.74) | 0.765 |
| *Citobacter spp.* | 5(1.7) | 6(4.44) | 1.62(.067-39.38) | 0.767 |
| *MRSA* | 28(8.7) | 14(10.44) | 0.113(0.01-1.081) | 0.058 |
| *Enterococcus Spp.* | 31(9.0) | 09(6.71) | 0.316(.032-3.126) | 0.324 |
| SSI | 150(43.3) | 39(7.73) | 0.424(.253-.709) | 0.001 |
| UTI | 111(32.0) | 75(14.88) | 0.20(0.11-0.38) | <0.001 |
| BSI | 41(10.7) | 43(8.5) | 0.53(0.25-1.14) | 0.15 |
| Pneumonia | 39(0.3) | 16(3.17) | 0.062(0.006-0.618) | 0.018 |
| VAP | 2(9.7) | 09(1.7) | 2.350(0.757-3.25) | 0.032 |
| Polymicrobial | 52(17.3) | 37(43.4) | 1.58(0.36-2.35) | 0.027 |
| Community-acquired  Nosocomial | 64(21.3)  227(75.7) | 46(25.3)  136(74.7) | 1.53(0.49-4.7)  1.39(0.50-4.04 | 0.012  0.031 |
| GNB  GPB | 257(66.23)  80(68.96) | 131(33.77)  36(31.04) | 1.53(0.49-4.7)  1.39(0.50-4.04 | 0.54  0.39 |
| MDR  XDR | 177(35.11)  66(13.09) | 65(12.89)  84(16.66) | 3.40(2.12-5.44)  8.12(4.11-16.06) | <0.001*  <0.001* |
